# The first deep-snouted tyrannosaur from Upper Cretaceous Ganzhou City of southeastern China

**DOI:** 10.1038/s41598-024-66278-5

**Published:** 2024-07-25

**Authors:** Wenjie Zheng, Xingsheng Jin, Junfang Xie, Tianming Du

**Affiliations:** Zhejiang Museum of Natural History, Hangzhou, 310014 Zhejiang People’s Republic of China

**Keywords:** *Asiatyrannus xui*, Tyrannosauridae, Nanxiong Formation, Upper Cretaceous, China, Palaeontology, Taxonomy

## Abstract

Tyrannosaurids were the most derived group of Tyrannosauroidea and are characterized by having two body plans: gracile, long-snouted and robust, deep-snouted skulls. Both groups lived sympatrically in central Asia. Here, we report a new deep-snouted tyrannosaurid, *Asiatyrannus xui* gen. et sp. nov., from the Upper Cretaceous of Ganzhou City, southeastern China, which has produced the large-bodied and long-snouted *Qianzhousaurus*. Based on histological analysis, the holotype of *Asiatyrannus xui* is not a somatically mature adult, but it already passed through the most rapid growth stages. *Asiatyrannus* is a small to medium-sized tyrannosaurine, with a skull length of 47.5 cm and an estimated total body length of 3.5–4 m; or around half the size of *Qianzhousaurus* and other large-bodied tyrannosaurines in similar growth stages. *Asiatyrannus* and *Qianzhousaurus* are sympatric tyrannosaurid genera in the Maastrichtian of southeastern China. *Asiatyrannus* differs from *Qianzhousaurus* in that it has a proportionally deeper snout, longer premaxilla, deeper maxilla, and deeper dentary, and the cornual process of the lacrimal is inflated without developing a discrete horn. The different skull proportions and body sizes suggest that *Asiatyrannus* and *Qianzhousaurus* likely had different feeding strategies and occupied different ecological niches.

## Introduction

Tyrannosauroids are the most distinctive, best known, and most intensively studied groups of dinosaurs, and are represented by almost thirty species^1–5^. The earliest tyrannosaurs appeared in the Middle Jurassic, around 165 million years ago^4,6–8^. They became the apex predators in their respective ecologies during the final 20 million years of the Cretaceous in Asia and western North America^9^. The colossal body size and deep snout are the characteristics of the ecologically dominant latest Cretaceous tyrannosaurid species^9^.

The tyrannosaurs in Asia are predominantly found in northern China and Mongolia, with the exception being the long-snouted genus *Qianzhousaurus* from the Nanxiong Formation (Maastrichtian) in Nankang District of Ganzhou City, southeastern China^10^. The Nanxiong Formation, which is distributed in Jiangxi and Guangdong provinces, is extremely fossiliferous with a large quantity of dinosaur skeletal remains and dinosaur eggs^10–12^. However, the theropods of the Nanxiong Formation are almost exclusively oviraptorosaurs, with more than seven species of oviraptorosaurs having been reported^12–16^ with *Qianzhousaurus* being the only non-oviraptorosaur theropod from the Nanxiong Formation yet described. Currently, *Qianzhousaurus* is retrieved as belonging to a clade of long-snouted Alioramini within the derived clade of Tyrannosauridae^10^. Beyond *Qianzhousaurus*, only some isolated theropod teeth, including possible tyrannosaurid teeth, had been reported from the Nanxiong Formation^17^. Other than that, several large tyrannosauroid teeth had been reported from the Maastrichtian Dalangshan Formation, Guangdong Province^18^. The coexistence of the long-snouted *Alioramus* and the deep-snouted *Tarbosaurus* are found together at the Tsaagan Khuushu locality in Mongolia, demonstrating that multiple large tyrannosaurids were able to live in sympatry within Asia^19^.

Here, we report the discovery of a new deep-snouted tyrannosaurid from the Upper Cretaceous of Ganzhou City, southeastern China. This new tyrannosaurid specimen was found in a construction site in Shahe Town, Nankang District, Ganzhou City, in September 2017. The specimen was prepared by technicians Chaohe Yu and Anhao Liu in the Zhejiang Museum of Natural History preparation laboratory, Hangzhou, China (ZMNH).

## Results

### Systematic paleontology

Theropoda Marsh, 1881

Tetanurae Gauthier, 1986

Coelurosauria Huene, 1914

Tyrannosauroidea Osborn, 1905

Tyrannosauridae Osborn, 1905

Tyrannosaurinae Osborn, 1905

*Asiatyrannus xui* gen. et sp. nov.

#### Etymology

The generic name is derived from Asia, and the suffix ‘tyrannus’ is derived from the Latin word for ‘king’ or ‘tyrant’, to emphasize that this is the new tyrannosaur collected in the continent of Asia. The specific name honors Dr. Xing Xu (Institute of Vertebrate Paleontology and Paleoanthropology, Chinese Academy of Sciences), a distinguished dinosaurologist who contributed greatly to the study of dinosaurs from China, including the research of several tyrannosaurs: *Guanlong*, *Dilong*, and *Yutyrannus*^20–22^. Dr. Xing Xu has also been a great supporter of the paleontological research and science popularization work of the Zhejiang Museum of Natural History.

#### Holotype

**Figure 1:**
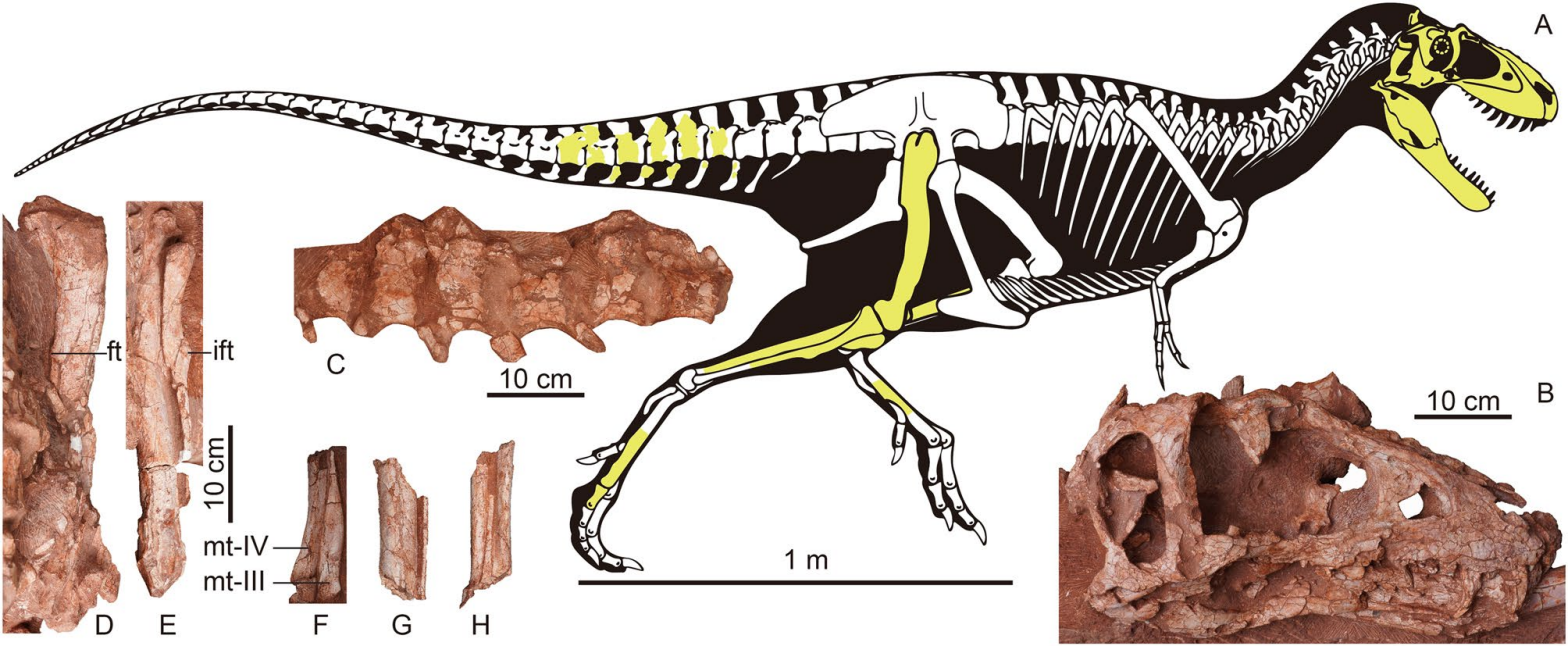
Fossil remains of *Asiatyrannus xui* (ZMNH M30360). (**A**) Skeletal outlines showing recovered elements in yellow color. The skeletal reconstruction is the proportional scaling of *Lythronax argestes* drawn by Scott Hartman from Loewen et al.^23^; (**B**) the skull in right lateral view; (**C**) the caudal vertebrae in left lateral view; (**D**) the right femur in posterior view; (**E**) the right tibia and fibula in posterior view; (**F**) The distal portion of the right metatarsal III in medial view and metatarsal IV in anterior view; (**G**) the middle shaft of the left tibia and fibula in anterior view; (**H**) The middle shaft of the left metatarsals in anterior view. *ft* the fourth trochanter; *ift* iliofibularis tubercle; *mt* metatarsal.

**Figure 2:**
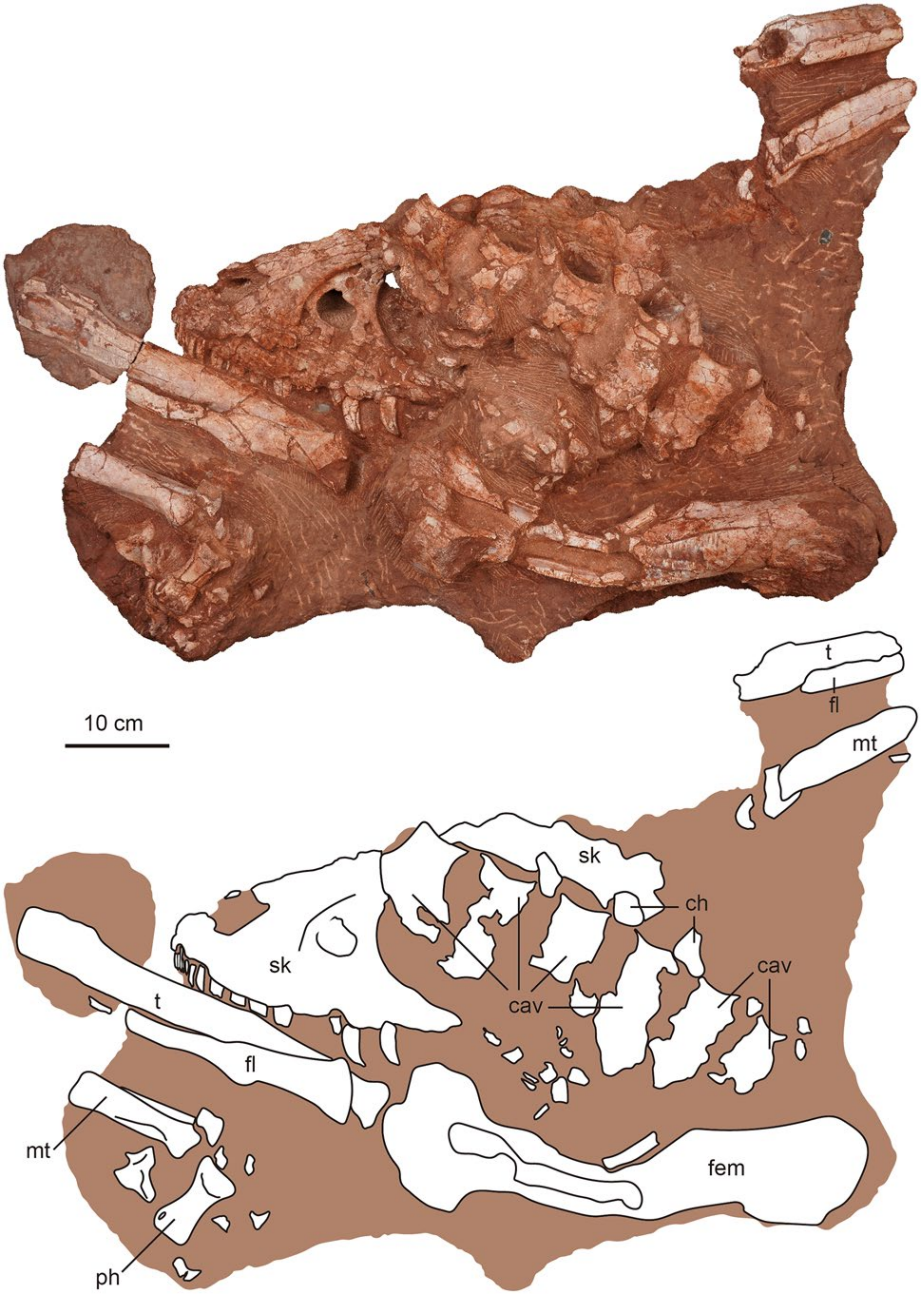
The photography and line drawing of the holotype of *Asiatyrannus xui* (ZMNH M30360). *cav* caudal vertebra; *ch* chevron; *fem* femur; *fl* fibula; *mt* metatarsal; *ph* phalanx; *sk* skull; *t* tibia.

**Figure 3:**
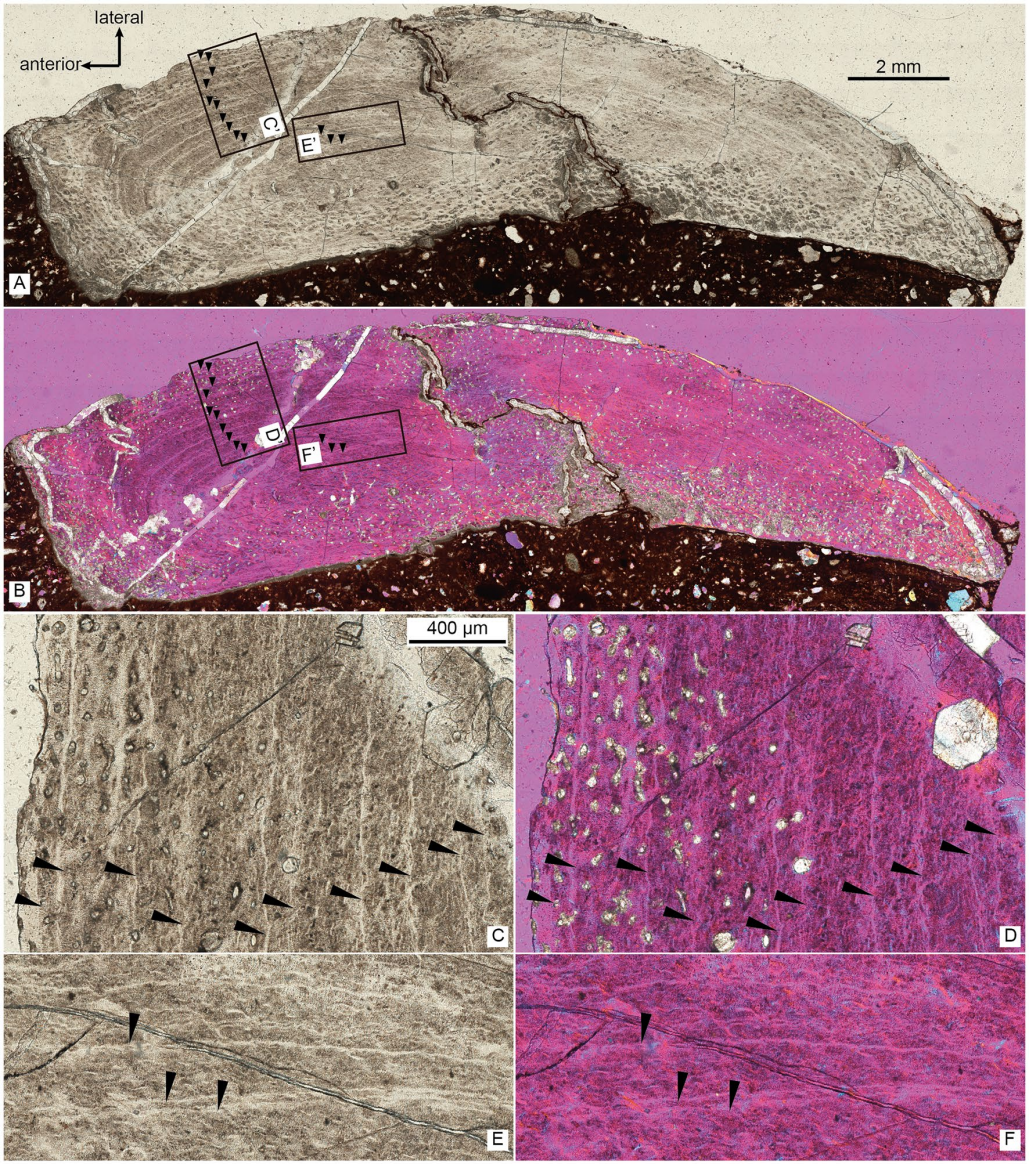
The thin section of the right fibula of *Asiatyrannus xui* (ZMNH M30360) in normal light (**A**) and cross-polarized light using a lambda compensator (**B**), inset boxes C’–F’ indicate the position of the detailed pictures in (**C**–**F)**; the detail of right fibula in normal light (**C**, **E**), and in cross-polarized using a lambda compensator (**D**, **F**). Black arrows denote lines of arrested growth (LAGs).

**Figure 4:**
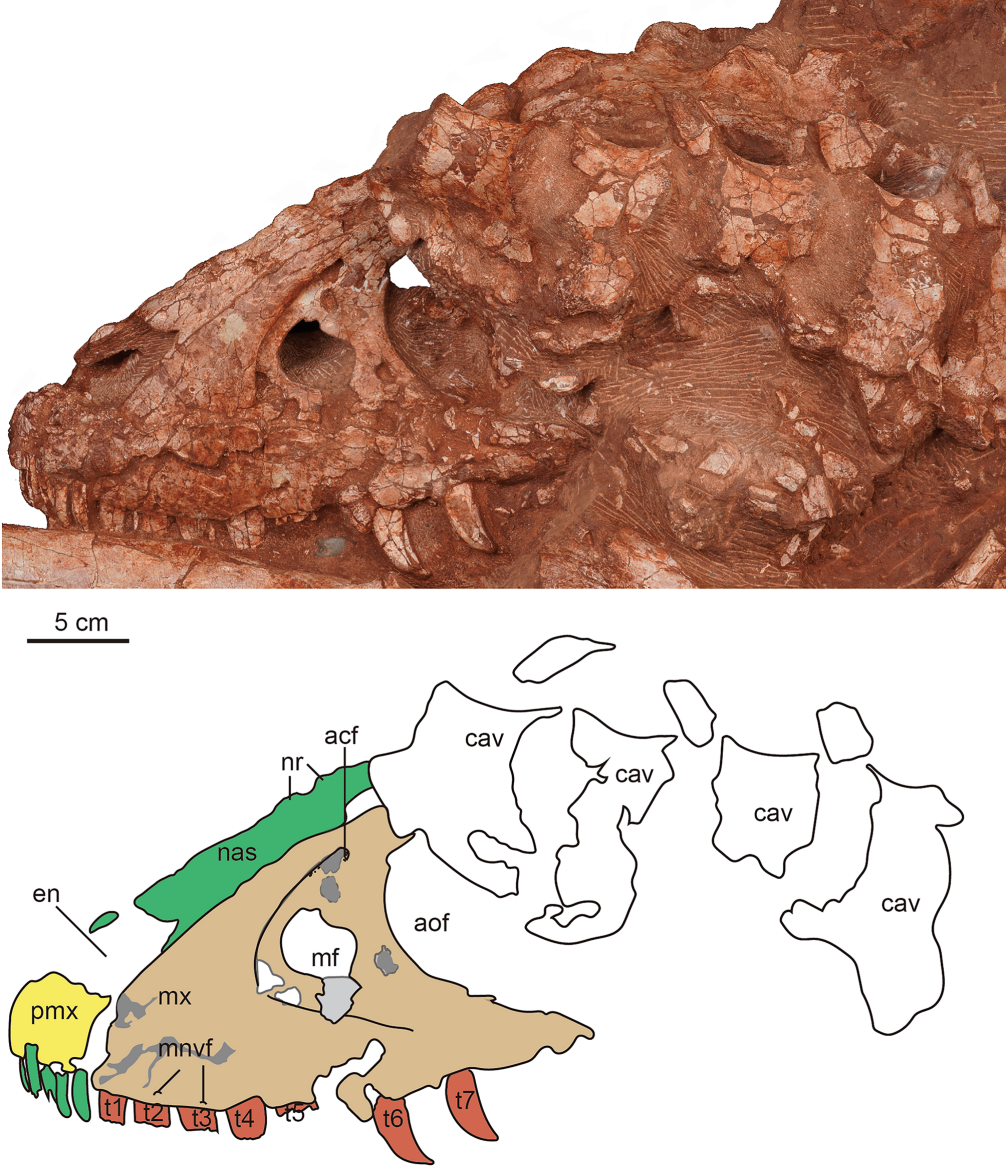
The photograph and line drawing of the skull of *Asiatyrannus xui* (ZMNH M30360) in left lateral view. *acf* accessory fossa of maxilla; *aof* antorbital fenestra; *cav* caudal vertebra; *en* external naris; *mf* maxillary fenestra; *mnvf* maxillary neurovascular foramina; *mx* maxilla; *nas* nasal; *nr* nasal ridge; *pmf* promaxillary fenestra; *pmx* premaxilla; *t1–7* maxillary tooth 1–7.

**Figure 5:**
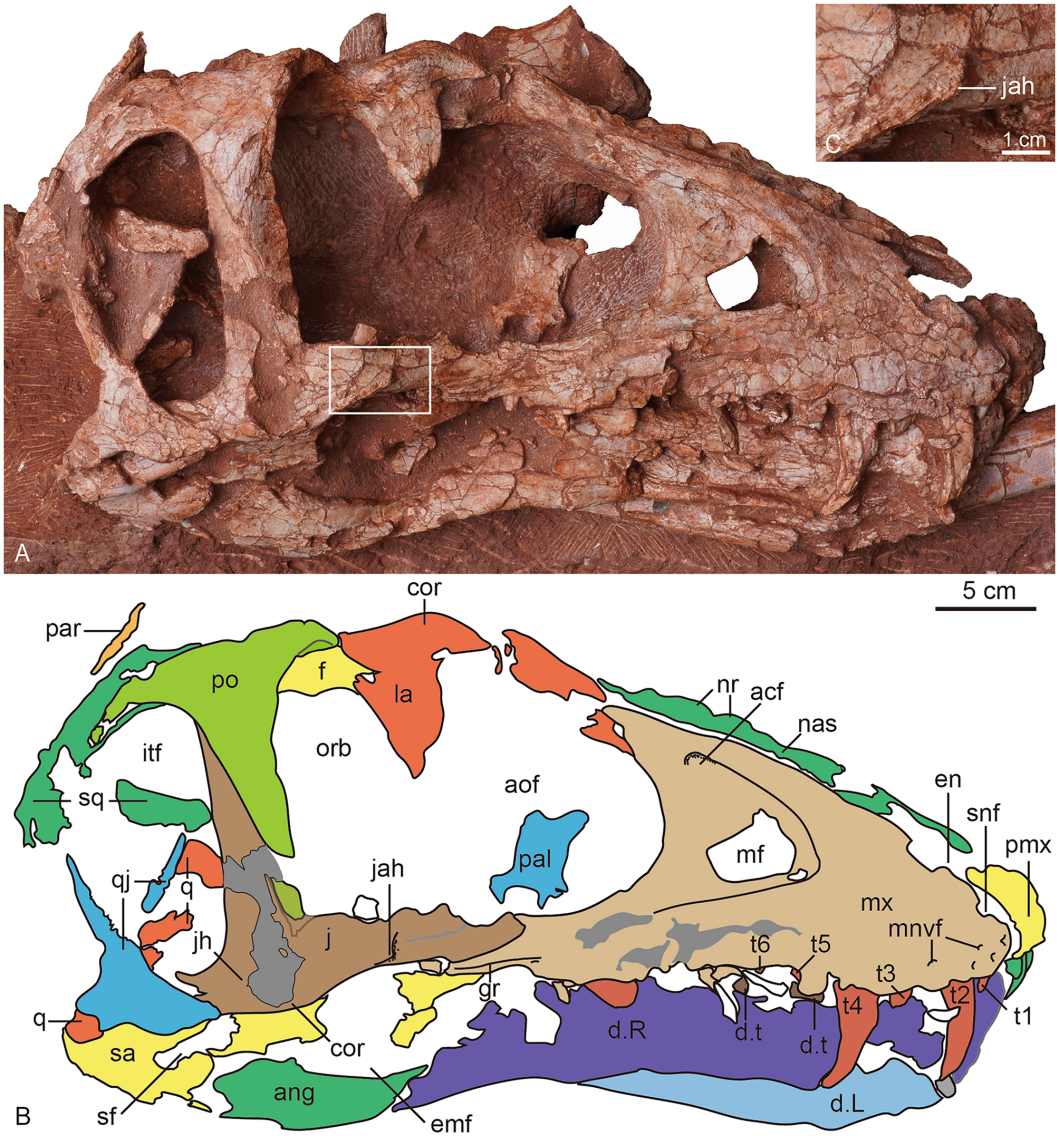
The photograph (**A**) and line drawing (**B**) of the skull of *Asiatyrannus xui* (ZMNH M30360) in right lateral view. The inset box in (**A**) indicates the position of the detailed jugal accessory horn in (**C**). *acf* accessory fossa of maxilla; *ang* angular; *aof* antorbital fenestra; *cor* cornual process; *d* dentary; *d.t* dentary tooth; *emf* external mandibular fenestra; *en* external naris; *f* frontal; *gr* groove; *itf* infratemporal fenestra; *j* jugal; *jah* jugal accessory horn; *L* left; *la* lacrimal; *mf* maxillary fenestra; *mnvf* maxillary neurovascular foramina; *mx* maxilla; *nas* nasal; *nr* nasal ridge; *orb* orbit; *par* parietal; *pal* palatine; *pmx* premaxilla; *po* postorbital; *q* quadrate; *qj* quadratojugal; *R* right; *sa* surangular; *sf* surangular foramen; *snf* subnarial foramen; *sq* squamosal; *t1–6* maxillary tooth 1–6.

**Figure 6:**
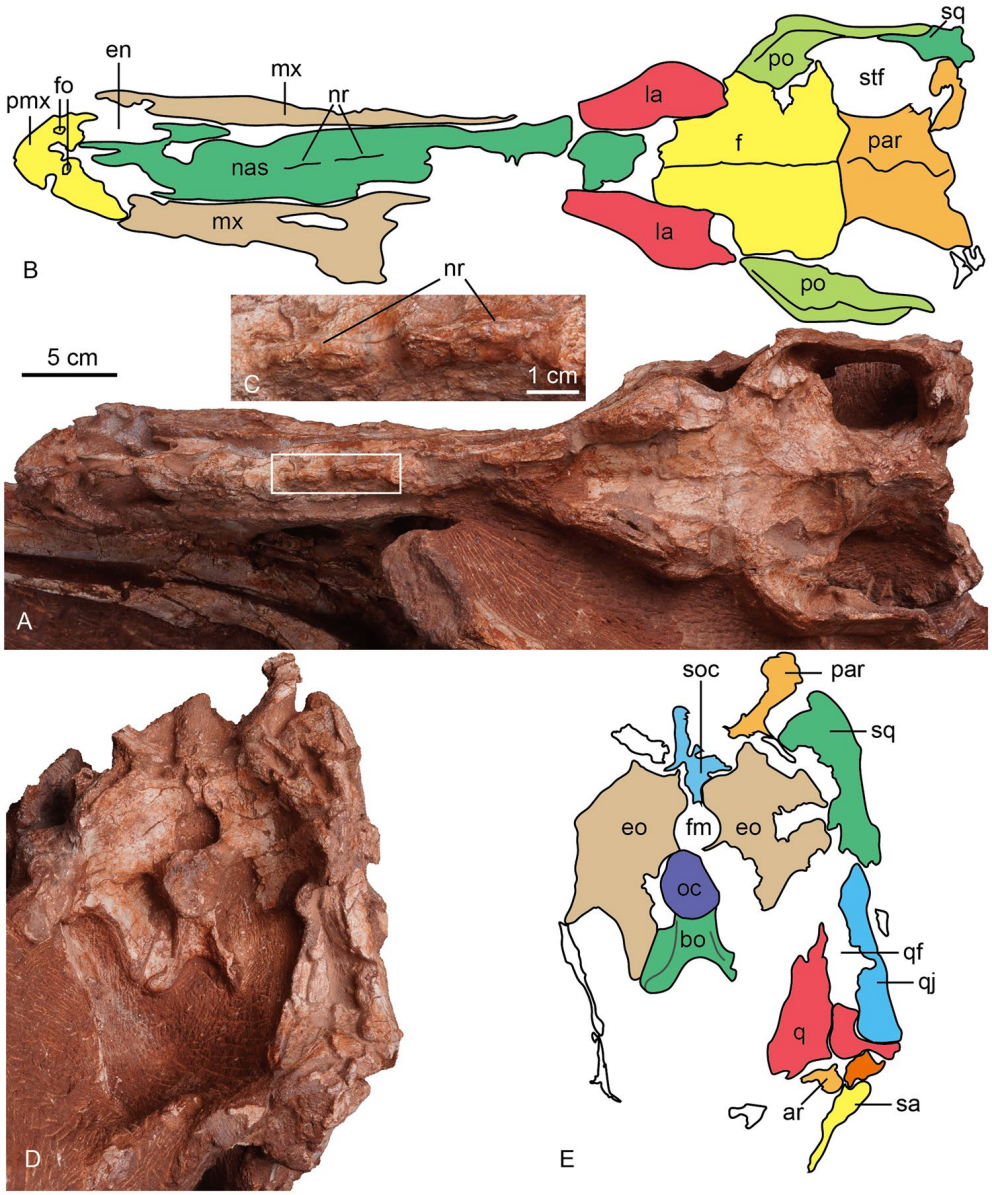
The photograph and line drawing of the skull of *Asiatyrannus xui* (ZMNH M30360) in dorsal (**A**, **B**) and posterior (**D**, **E**) views. The inset box in (**A**) indicates the position of the detailed medium ridges of the nasal in (**C**). *ar* articular; *bo* basioccipital; *en* external naris; *eo* exoccipital; *fm* foramen magnum; *fo* fossa; *la* lacrimal; *mx* maxilla; *nr* nasal ridge; *nas*, nasal; *oc* occipital condyle; *par* parietal; *pmx* premaxilla; *po* postorbital; *q* quadrate; *qf* quadrate foramen; *qj* quadratojugal; *sa* surangular *soc* supraoccipital; *sq* squamosal; *stf* supratemporal fenestra.

**Figure 7:**
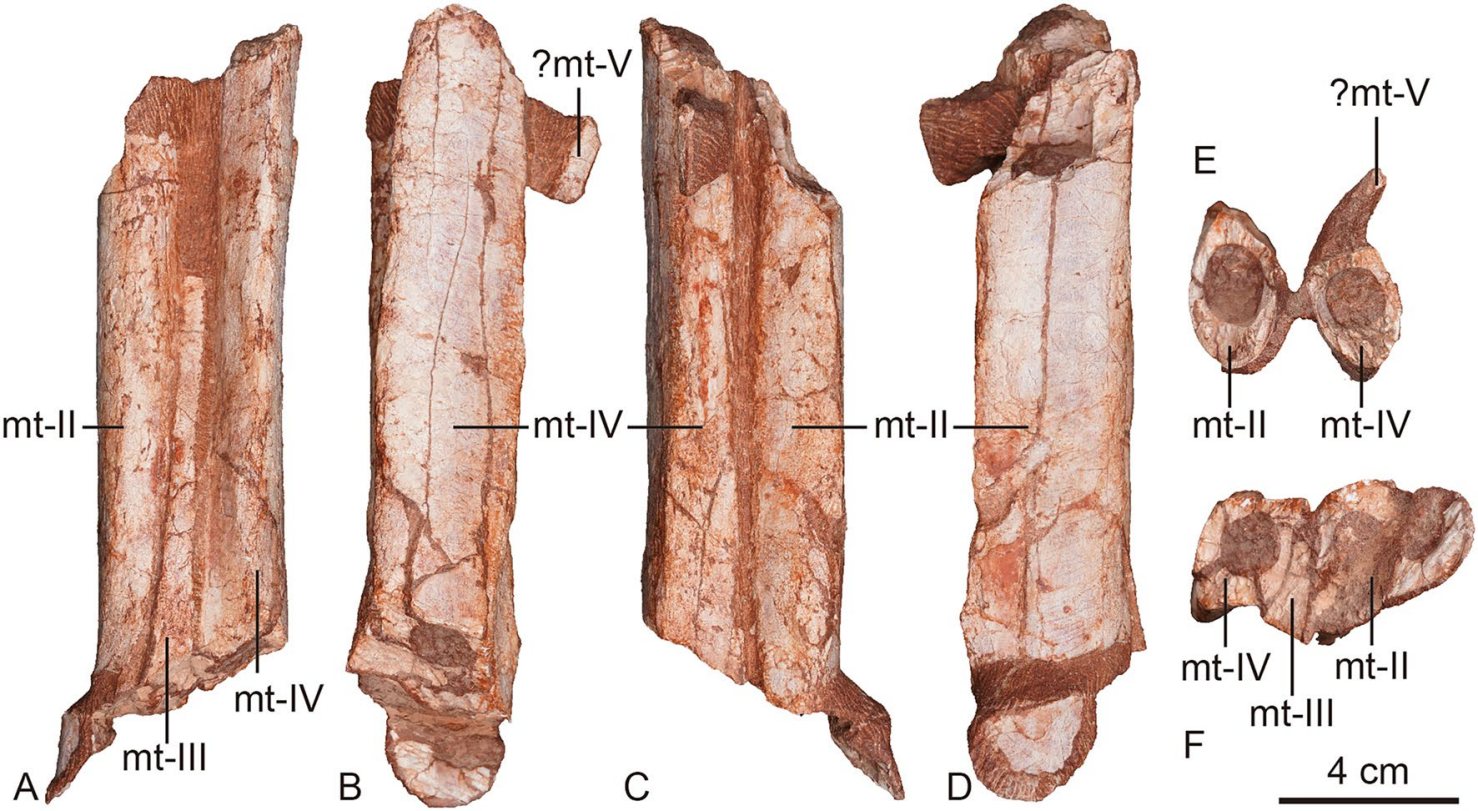
The left metatarsals *Asiatyrannus xui* (ZMNH M30360) in anterior (**A**), lateral (**B**), posterior (**C**), medial (**D**), proximal (**E**), and distal (**F**) views. *mt* metatarsal.

ZMNH M30360, housed at Zhejiang Museum of Natural History, Hangzhou, Zhejiang, China. A nearly complete skull and partial disarticulated postcranial skeleton, including caudal vertebrae; right femur, tibia, and fibula; metatarsals, pedal phalanges, and a partial midshaft of the left tibia, fibula, and metatarsals (Figs. 1, 2, 3, 4, 5, 6 and 7, Table 1).

**Table 1:** Selected measurements of the holotype of *Asiatyrannus xui* (ZMNH M30360). The asterisk indicates a measurement from an incomplete element due to damage.

Element	Measurement (cm)
Skull length (premaxilla-occipital condyle)	47.5
Premaxilla height, body ventral to naris, left	3.8
Premaxilla height, body ventral to naris, right	3.5
Premaxilla body length, left	3.3
Premaxilla body length, right	3.0
Maxilla preserved length* right	29.2
Maxilla preserved height* right	13.2
Maxilla dorsoventral depth at the anterior margin of the antorbital fenestra, right	12.0
Femur length, right	44.0
Tibia length, right	40.5*

#### Type locality

Nanxiong Formation (Upper Cretaceous, Maastrichtian) of Shahe Town, Nankang District, Ganzhou City, Jiangxi Province, China.

#### Diagnosis

*Asiatyrannus xui* is a small to medium-sized tyrannosaurine theropod that differs from other tyrannosauroids in possessing the following autapomorphies: two small, deep fossae located on the lateral surface of the premaxilla just lateral to the anteroventral border of the external naris, a large and sub-rectangular shaped maxillary fenestra, the posterior protuberances of the nasals connect to form two separated medium low ridges, a low ridge-like jugal accessory horn, the ventral margin of the anterior ramus of the jugal curving ventrally anterior to the accessory horn, the lateral surface of the descending process of the postorbital developed the anterodorsally trending fine lineations, the slender, straight, and banded-shaped postorbital bar, with almost straight and parallel anterior and posterior margins in lateral view, and the lateral surangular shelf extends to the posterior end of the surangular.

### Ontogenetic age and body size

The periosteal cortex of the right fibula is zonal and exhibits a minimum of 13 lines of arrested growth (LAG) (Fig. 3). The fibular section lacks a medullary cavity similar to the juvenile *Tarbosaurus* MPC-D 107/7^24^, however, the secondary remodeling is more developed in ZMNH M30360 than MPC-D 107/7^24^. The periosteal bone is incomplete; therefore, the total number of LAGs and the presence of an external fundamental system cannot be determined. Thus, the *Asiatyrannus* had a terminal age of at least 13 years of life. The outer zones became narrower, indicating a slowdown of growth was occurring, but the animal was still experiencing active growth. The medial half of the fibula had undergone a large degree of secondary remodeling, which obliterated the LAGs (Fig. 3). The narrower outer zone and the highly developed secondary osteons indicate that the holotype of the *Asiatyrannus xui* is not a full-growth adult individual, but it already passed through the most rapid growth stage. The skull of *Asiatyrannus* shows several matured cranial morphology: the ventral edge of the maxilla has a distinct convex curvature; the nasals are fully fused with pronounced vaulting and well development of protuberances; the cornual process of lacrimal is strongly inflated similar to the adult *Tyrannosaurus* and *Tarbosaurus*^2^; the dentary is relatively deep. Thus, we interpret the specimen to be a sub-adult that was nearing somatic maturity. The early branching, small-bodied tyrannosauroid *Guanlong* and *Moros* reached or neared their adult size at 6–7 years of age^20,25^. By contrast, the large-bodied, Campano-Maastrichtian tyrannosaurines were undergoing exponential stage growth around 14 years old^26^. By comparison, the *Asiatyrannus* holotype is not a full-growth individual and experienced an earlier exponential growth rate when compared with other large-bodied tyrannosaurines from North America^26^. The holotype’s femoral length is around half the length of individuals with a similar number of LAGs^26^, thus indicating *Asiatyrannus* represents a relatively small-bodied tyrannosaurine compared with other large-bodied Campano-Maastrichtian tyrannosaurines. The length of *Asiatyrannus* skull and femur is 47.5 cm and 44 cm, respectively. The total body length of *Asiatyrannus* is estimated from 3.5 to 4 m (Fig. 1). The skull is about 8% longer than the femur, while the same ratio in *Qianzhousaurus* is 29% since the length of the skull and femur is 90 cm and 70 cm, respectively^10^.

### Comparative description

The holotype of the *Asiatyrannus xui* preserves a nearly complete skull and partial postcranial skeleton. The right side of the skull is well-exposed, but the posterior half of the left side is overlain by the postcranial skeleton. The external texture of most bones is not well-preserved, with the periosteal surface obscured by cracks, breaks, holes, and fractures, resulting from taphonomic processes.

#### Skull

The posterior half of the left skull is obscured by the caudal vertebrae, and the left mandible is concealed by the hindlimb (Figs. 2, 4), the right side of the skull is well-exposed (Fig. 5). Overall, the skull is well-preserved with all bones articulated with each other, however, some sutures between the bones are difficult to observe due to breakage or poor surface preservation (Figs. 2, 4, 5 and 6). The skull is deeper dorsoventrally than it is transversely wide as in other tyrannosaurids^2^. The lateral profile of the skull is deeper than alioramins *Alioramus*^27,28^, and *Qianzhousaurus*^10^, and is similar to other large-bodied tyrannosaurids like *Tarbosaurus*, *Tyrannosaurus*, and *Gorgosaurus*^2^. The snout and the skull are both relatively narrower than that in large-bodied tyrannosaurids *such as Gorgosaurus*^2^, *Tarbosaurus*^29^, and *Tyrannosaurus*^29,30^. The length of the skull is 47.5 cm from the anterior tip of the premaxilla to the posteriormost point of the occipital condyle, much smaller—but proportionally deeper—than that of *Qianzhousaurus*, which is 90 cm in length^10^. In tyrannosaurids, the skull becomes deeper and more robust with increasing maturity or size^2,22^.

#### Premaxilla

Both premaxillae are preserved though both the ascending processes are damaged, and the outer surfaces of the premaxillae are broken in areas. The ventral ramus of the premaxilla is taller dorsoventrally than it is long anteroposteriorly, as in other tyrannosauroids^2^. The length of the ventral surface of the premaxilla is around 6.3–6.9% of the overall basal skull length (Table 1), which is similar to ∼4–10% of other tyrannosauroids, except extremely low 2.2% of *Qianzhousaurus*^10,31^. The anterior margin of the premaxilla is oriented almost vertically in lateral view, similar to *Tarbosaurus*^29^, and *Tyrannosaurus*^32^. This same margin is sloped posterodorsally in ‘*Nanotyrannus*’^1^. The premaxilla forms the anterior and ventral edge of the external naris, which is anteroposteriorly long with its long axis sloping anteriorly. Two small, deep fossae are located on the lateral surface of the premaxilla just lateral to the anteroventral border of the external naris (Fig. 6). The fossae, which are present on both premaxillae, are not seen in other tyrannosaurs. Four conical, elongated premaxillary teeth are considerably smaller than the maxillary teeth, as in *Xiongguanlong*, *Timurlengia*, and other Late Cretaceous tyrannosauroids^2,33^. All premaxillary teeth are visible in anterior view, as is characteristic of tyrannosaurids^10^. Two small foramina are preserved along the ventral margin of the right premaxilla parallel to the tooth row (Fig. 5). The premaxilla is well-separated from the maxilla in lateral view, likely due to the taphonomic distortion, so the location of the subnarial foramen is unclear.

#### Maxilla

Both maxillae are well-preserved. The ventral margin of the right maxillae is incomplete. The better preserved left maxilla has a distinct and ventrally oriented convex curvature near its midpoint, as seen in *Timurlengia* and Late Cretaceous large-bodied tyrannosauroids^2,32,33^, contra to the straight or slightly convex in early-branching tyrannosauroids, such as *Guanlong*^20^, *Dilong*^21^, *Xiongguanlong*^34^, and *Suskityrannus*^33^. The curvature of the ventral margin is an ontogenetic feature with this margin having a much weaker convexity in juveniles than in adults, as is seen in *Tarbosaurus*^24,29,35^. The curvature of the ventral margin is more strong than in juvenile *Tarbosaurus*^24^, but as strong as in large-bodied adult tyrannosauroids, such as *Tarbosaurus*^29^, and *Tyrannosaurus*^32^. The main body of the maxilla is deep and short as in other large adult tyrannosaurids such as *Tyrannosaurus* and *Tarbosaurus*^19^. Contra with the long and low maxilla in *Alioramus remotus*^28^, *Alioramus altai*^19^, and *Qianzhousaurus*^10^. The promaxillary fenestra is obscured in lateral view, it rotates to face anteriorly as in other tyrannosaurines^2^. Unlike other tyrannosaurines, the anterior margin of the maxillary fenestra does not contact the anterior rim of the antorbital fossa^2^. The main body of the maxillae tapers in depth posteriorly below the antorbital fenestra.

Although the outer surface of the maxillae is full of cracks, it is clear the surface does not develop the dorsoventrally trending grooves and ridges present on other tyrannosaurids, as is the case in adult *Daspletosaurus* (CMN 8506), *Tarbosaurus*, *Tyrannosaurus*^19^, and *Zhuchengtyrannus*^36^ specimens. The dorsoventrally trending grooves are present both in juvenile and adult *Tarbosaurus*^24,29^. Anteriorly, the maxillae articulate with the premaxilla, and the contact margin is oriented dorsoventrally resulting in a rounded anteroventral corner, as in most tyrannosaurids^19^. This condition differs from the strong posterodorsal orientation of the premaxillary articulation margin and sharp anteroventral point in *Alioramus remotus*^28^, *Alioramus altai*^19^, and *Qianzhousaurus*^10^. Posteriorly, the main body of the maxilla is articulated with the jugal, with the dorsal half of the maxilla overlaid by the jugal. Some alveolar neurovascular foramina are preserved immediately dorsal and parallel to the tooth row. The antorbital fossa is extensive and extends beyond the antorbital fenestra anteriorly and ventrally, similar to the condition found in *Alioramus altai*^19^, and *Appalachiosaurus*^37^. However, this condition differs from large adult tyrannosaurids, such as *Tyrannosaurus* and *Tarbosaurus*, where there is little lateral exposure of the fossa below the antorbital fenestra^19^.

The maxillary fenestra is large and sub-rectangular in shape, similar to the adult *Tarbosaurus*^29^, in contrast, the fenestra is oval in juvenile *Tarbosaurus*^24^. This fenestra is located relatively anteriorly between the anterior edge of the antorbital fossa and fenestra. In contrast, the fenestra is located more centrally along the maxilla of *Alioramus altai*, *Appalachiosaurus*, *Albertosaurus*, *Bistahieversor*, *Gorgosaurus*, and juvenile tyrannosaurines^19^. The margin of the maxillary fenestra is widely separated from that of the external antorbital fenestra, as in small-bodied tyrannosauroids such as *Raptorex*^38^, and juvenile specimens of tyrannosaurids, like *Tarbosaurus*^24^. Although the skull is smaller, the fenestra is relatively larger than in *Appalachiosaurus*^37^, juvenile and adult *Gorgosaurus*^39,40^, and ‘*Nanotyrannus*’^1^. The maxillary tooth row is not completely preserved. The first tooth of the right maxilla is small and incisiform, similar to the premaxillary teeth (Fig. 5), but the first tooth of the left maxilla seems as large as the second one (Fig. 4).

#### Nasal

The left and right nasals are fused medially into a single vaulted element, a characteristic feature of tyrannosauroids^19,41^. Anteriorly each nasal divides into two processes: the subnarial process and the premaxillary process, as in other tyrannosaurs^19^. Anteriorly, the premaxillary processes are separated on the midline. The nasal is excluded from the antorbital fossa by the maxilla and lacrimal, in contrast, the nasal forms a part of this margin in adult *Tarbosaurus*^24,29^. In dorsal view, the subnarial processes are lateral to the premaxillary processes. This condition is also seen in *Gorgosaurus*, *Albertosaurus*, some specimens of *Tyrannosaurus* (BHI 3033), and *Alioramus altai*^19^. In contrast, the subnarial process is nearly beneath the premaxillary process in *Daspletosaurus* (CMN 8506) and other specimens of *Tyrannosaurus* (CM 79057)^19^. The nasals don’t show any separation in the anterior end. However, they are separated at the anterior and posterior ends in juvenile and adult *Tarbosaurus*^24^. The dorsal margin is straight in lateral view, in contrast, the anterior half of the dorsal margin is vaulting in lateral view in adult *Tarbosaurus*^29^.

The nasal has pronounced vaulting observed in large tyrannosaurids^42^. The dorsal external surface of the nasal is rugose with prominent developed nasal protuberances, as is the case in most other tyrannosaurids^2,32^. The protuberances are homologous to the rugosities seen in all Late Cretaceous tyrannosauroids, *Alioramus remotus*^28^, *Appalachiosaurus*^37^, *Yutyrannus*^22,34^, except *Xiongguanlong*^34^. The rugose region is developed in all preserved nasals and extends around the middle of the antorbital fenestra. The posterior protuberances are connected to form two successive separated low medium ridges (Figs. 4, 5 and 6: nr). The development of the nasal protuberances is an ontogenetic feature and the nasal becomes more pronounced in larger specimens^2^. The dorsal surface of the nasal is smooth in *Raptorex*^38^, and the juvenile *Tarbosaurus*^24^. The pronounced protuberances on the fused nasal of *Asiatyrannus* are similar to other adult tyrannosaurids, such as *Tarbosaurus*^29^, *Tyrannosaurus*^32^, and *Qianzhousaurus*^10^.

#### Lacrimal

Both both lacrimals are preserved, but the caudal vertebrae obscure the left and the right is incomplete (Figs. 5, 6). The bone is L-shaped in lateral view, with a short posterior process that is similar to that of *Tyrannosaurus*^1^. The angle between the anterior and ventral rami is acute, similar to *Daspletosaurus*, *Tarbosaurus*, *Tyrannosaurus*, and *Alioramus altai*^19^. The cornual process is inflated without a discrete hornlet similar to the *Tyrannosaurus*-*Tarbosaurus* clade^2^. The cornual process is inflated similar to that of *Tyrannosaurus*^32^, and is more inflated than in *Appalachiosaurus*^37^, and the adult specimen of *Tarbosaurus*^19,29^. However, the surface of the cornual process is smoother than that of *Tarbosaurus*^29^. The inflation is not developed in *Raptorex*^38^, or less pronounced in the juvenile specimen of *Tarbosaurus*^24^. The cornual process has a triangular horn in *Yutyrannus*^22^, albertosaurines^2,40^, *Daspletosaurus*^2^, *Alioramus altai*^19^, and *Qianzhousaurus*^31^.

#### Jugal

The lineation between the postorbital and jugal is a straight, obliquely angled line. This contact divides the postorbital bar diagonally in an anteroventral to posterodorsal direction. However, the line is distinctly bent in *Daspletosaurus*, *Albertosaurus*^2^, and *Tarbosaurus*^29^. The ascending process terminates at the anterodorsal rim of the infratemporal fenestra but does not contact the squamosal. The ascending process meets the squamosal and excludes the postorbital from the infratemporal fenestra anteriorly in the *Tyrannosaurus*^32^. The lateral surface of the ascending process is smooth, as in *Albertosaurus*, *Bistahieversor*, *Gorgosaurus*, *Teratophoneus*, juvenile tyrannosaurines, and *Alioramus altai*^19^. By contrast, this lateral surface is excavated by a broad concavity in large subadult and adult tyrannosaurines (*Daspletosaurus*, *Tarbosaurus*, *Tyrannosaurus*)^19^. The entire postorbital contact zone is demarcated posteriorly by a pronounced ridge, as in *Alioramus altai*^19^. The ridge is especially prominent in the dorsal half of the jugal.

Posterior to the ascending process, the jugal forms the ventral floor of the infratemporal fenestra. The posterior ascending process of the jugal has an acute dorsal tip and is overlapped by the postorbital anteriorly, as in *Tyrannosaurus*^32^. The jugal horn appears to be developed, but the broken surface of the horn makes the condition uncertain. The jugal horn is only well developed in *Alioramus altai*^19^, and ridge-like in *Qianzhousaurus*^31^. Similar to *Alioramus altai*^19^, the lateral surface of the jugal’s external surface develops anteroposterior raised lineations. Along the ventral margin of the anterior ramus and ventral to the orbit, there is a ridge-like jugal accessory horn in the ventrolateral portion of the anterior process (Fig. 5). The accessory horn is not present in other tyrannosauroids including *Raptorex*^38^, the juvenile and adult of *Tarbosaurus*^24,29^. Anteriorly, the ventral margin of the anterior ramus has a ventral curvature anterior to the accessory horn. However, the anterior ventral margin is straight or curves slightly dorsally in other tyrannosauroids^2,30^, including juvenile and adult *Tarbosaurus*^24,29^.

#### Frontal

Both frontals are well-preserved (Figs. 5, 6). The frontals are sutured medially as in other tyrannosaurids^2^. In dorsal view, the contact with the postorbital is V-shaped, with a concavity located on the frontal, as in *Tyrannosaurus*^32^. However, the border between the prefrontal and the prefrontal is unclear. The posterior portion of the frontals slopes ventrally into the supratemporal space from the anterior and medial supratemporal borders with a low sagittal crest extending posteriorly to parietals. The supratemporal fossae in the frontals are short, as in ’*Nanotyrannus*’, whereas they are elongated in *Tyrannosaurus*^1^. There is only a short contribution of frontal to the sagittal crest, like in ‘*Nanotyrannus*’. In contrast, there is a long frontal contribution to the sagittal crest in *Tyrannosaurus*^1^. The frontal has a small, notch-like orbital rim, as in later-diverging taxa^33^. In contrast, the frontal makes a wide contribution to the orbital rim in *Guanlong*, *Dilong*, and *Suskityrannus*^33^.

#### Parietal

As in other tyrannosaurids, the parietals possess a fused sagittal crest^2,32^. The nuchal crest is broken and not present along the parietals. The frontoparietal suture is distinct and transversely directed. The parietal slopes broadly laterally within the supratemporal space, forming the medial and medioposterior walls of the fenestrae.

#### Postorbital

The right postorbital is well-preserved and exposed, while the lateral surface of the left postorbital is obscured by caudal vertebrae. Although the anterior portion of the process is broken, the cornual process is swollen and is present as a rugose convex boss that is located posterodorsally to the orbit, as in mature specimens of all other tyrannosaurids^31^. This process is absent in basal tyrannosauroids, but present as well-developed, rugose bulges in tyrannosaurids^19^. The descending process is tongue-shaped and gracile, as in *Alioramus altai*^19^ and ‘*Nanotyrannus*’^1^. The descending process is straight and gracile, without an anteriorly expanded suborbital process. The descending process is anteroposteriorly broad and constricts the orbit in large individuals of *Yutyrannus*^22^, *Tarbosaurus*^29^, *Tyrannosaurus*^1^, and *Albertosaurus*^43^. The anterior expansion is incipient in the juvenile specimen of *Tarbosaurus*^24^, however, it is more conspicuous in the juvenile specimen of *Tarbosaurus* than in *Asiatyrannus*^24^. Although the anterior expansion is incipient in *Gorgosaurus*, the descending process curved anteriorly in juvenile and adult individuals^39,40^, in contrast, the anterior edge of *Asiatyrannus* is almost straight. The descending process reaches the floor of the orbit ventrally, however, the process terminated higher in juvenile and adult *Tarbosaurus*^24,29^. The lateral surface of the process has anterodorsally trending fine lineations. In contrast, the lineations are horizontally directed and dorsally convex in *Alioramus altai*^19^, *Albertosaurus*^39^, *Qianzhousaurus*^31^, *Tarbosaurus*^24^, and ‘*Nanotyrannus*’^1^. The postorbital bar composed of the jugal and postorbital is slender, straight, and banded-shaped, with almost straight and parallel anterior and posterior margins in lateral view. Generally, the postorbital bar is broader and the anterior margin is convex anteriorly due to the development of the anterior expansion of the postorbital. In other tyrannosauroids without anterior expansion development, the anterior margin is concave anteriorly, such as *Raptorex*^38^, *Daspletosaurus*^30^, ‘*Nanotyrannus*’^1^, and juvenile *Tarbosaurus*^24^.

#### Squamosal

The squamosal is broadly convex and slopes posteroventrally in lateral view. The anterolateral process is forked, with dorsal and ventral projections. The lateral surface is overlapped by the posterior ramus of the postorbital. In lateral view, the dorsal projection of the squamosal is exposed and is wider than the ventral projection. The descending process forms the derived squamosal-quadratojugal flange that bisects the infratemporal fenestra, which may be a tyrannosaurid synapomorphy^2,32,41^. The squamosal component contacts with the postorbital bar anteriorly and bisects the infratemporal fenestra completely, as in *Tyrannosaurus*^32^, but not in juvenile and adult *Gorgosaurus*^39,40^, juvenile and adult *Tarbosaurus*^24,29^, *Alioramus altai*^19^, and *Qianzhousaurus*^31^. The anterior tip of the flange is rugose and bulbous. The long axis of the ventral process projects roughly anteroposteriorly, as in other tyrannosaurids^19^. Posteroventrally, a portion of the squamosal is broken, however, the preserved portion of the posterior end directs posteroventrally. In the posterodorsal region of the lateral margin, there is a prominent thin ridge that extends along the dorsal edge. The crest directs dorsally and marks the highest point of the squamosal, as in *Tyrannosaurus*^32^. However, the ridge is located along the lateral surface of the squamosal rather than its dorsal edge as in *Tarbosaurus*^29^ and *Alioramus altai*^19^.

#### Quadratojugal

The anterior and posterior margins of the quadratojugal’s ascending processes are both concave, making the quadratojugal hourglass-shaped in lateral view. This condition is similar to that observed in other tyrannosaurids^19^. The posterior process is short and directs ventroposteriorly. In lateral view, the ventral edge of the quadratojugal is strongly concave.

#### Quadrate

The quadrate forms the mandibular condyle, which is transversely expanded. The condyle is extended posterior to the posteriormost extent of the paroccipital processes, contra to other tyrannosaurids^2^.

#### The occipital elements

The occipital condyle is incomplete. The sutural separation of the exoccipitals and basioccipital is only visible ventrally, lateral to the basal tubera. The paroccipital processes are broad. The basal tubera is deep and bilaterally narrow as in *Daspletosaurus* sp. (TMP 94.143.1)^39^, however, the basal tubera is broad in *Tyrannosaurus*^32^. The basal tubera is separated along its ventral margins by a concave notch that is deep and more than half of the depth of the tubera—similar to the derived tyrannosauroids^44^, *Alioramus remotus*^28^, and *Tarbosaurus*^29^. In contrast, this notch is shallow in *Tyrannosaurus*^32^, and *Qianzhousaurus*^31^, or narrow in ‘*Nanotyrannus*’^1^.

#### Mandible

The mandible is preserved in a closed position with the cranium, thus making the dorsal portion of the mandible obscured by the cranium. The mandible is robust and deep as in those of adult tyrannosaurids. The dentary is more robust and deeper dorsoventrally than those of alioramins, such as *Alioramus*^27^, and *Qianzhousaurus*^10^.

Both dentaries are preserved with most of the lateral right dentary and ventral portion of the medial left dentary exposed. The anterior edge of both dentaries is more vertically directed than those of alioramins, such as *Alioramus*^27^, and *Qianzhousaurus*^10^. The ventral edge of the dentary has only slight concave curvature, such as *Zhuchengtyrannus*^36^.

The surangular is badly damaged along the dentary and posterior margins of the external mandibular foramen. Dorsal to the surangular foramen, the lateral surangular shelf is prominent and overhangs its lateral surface, as in *Tyrannosaurus*^32^. The shelf extended to the posterior end. In contrast, the edge of the surangular ends posteriorly above the posterodorsal corner of the surangular foramen in ‘*Bagaraatan*’^45^, *Tyrannosaurus*^32^, and *Alioramus altai*^19^.

The angular is also badly damaged. The anterior dorsal edge, which borders the ventral of the external mandibular fenestra, appears complete and has a convex lateral surface^19^. The ventral edge of the angular forms the ventral edge of the mandible and is convex in lateral view. The ventral border of the angular is broadly convex, and there are no prominent rugosities as reported in *Tyrannosaurus*^19,32^.

#### Postcranial skeleton

##### Femur

The right hindlimb is well-preserved, with the femur, tibia, fibula, and partial pes preserved (Fig. 1). Only a partial tibia, fibula, and metatarsals from the left hindlimb were recovered (Figs. 2, 7). As in most theropods, including tyrannosauroids, the femur has a posteriorly oriented concave curve in lateral view (Fig. 2)^2,25^. The head of the femur projects mostly medially but also slightly proximally (Fig. 1). The proximal end of the right femoral head and the greater trochanter are partially missing due to the eroded nature of the proximal femur. Based on the preserved femur, the head is not distinctly elevated relative to the greater trochanter. Due to the incompleteness of the lesser trochanter, the relative height of the lesser and greater trochanters is indeterminate. They were separated by a deep, narrow cleft. The medullary cavity is expansive and cortical bone is relatively thin as in other tyrannosauroids^25^. In proximal view, the femoral head keeps a relatively constant width as it extends medially, similar to *Gorgosaurus* and *Tarbosaurus*^19^. A well-developed sulcus that would have housed ligaments is present along the midline of the posterior surface of the femoral head, as is observed in *Alioramus altai*^19^. The subtriangular fourth trochanter is powerfully developed along the posteromedial shaft, as in other tyrannosauroids^2,25^.

##### Tibia

Both tibiae are incomplete, with the right tibia being the most complete with only the distal articular condyles missing (Figs. 1, 2). The preserved length of the right tibia is shorter than the length of the right femur. Extensive hollowing is found throughout the tibia with the wall of the tibia being thin and the marrow cavity being relatively large.

##### Fibula

The fibular shaft is tightly appressed to the tibia as in *Bistahieversor*, and *Moros*^25^. The proximal half of the right fibula and the midshaft of the left fibula are preserved, with both articulating with the corresponding tibia (Figs. 1, 2). The iliofibularis tubercle is pronounced on the margin where the anterior and lateral surfaces meet, This iliofibularis tubercle, is large, prominent, rugose, and elongated proximodistally as in most tyrannosauroids like *Dryptosaurus*^46^. The fibular shaft is most constricted distal to the iliofiburlaris tubercle in lateral view.

##### Metatarsus and pes

The left and right metatarsals are preserved in articulation but are incomplete, with the mid-shaft section of the left metatarsal II–IV and the distal portion of right metatarsal III and IV being preserved (Figs. 1, 7). As in other tyrannosauroids, the metatarsus is long and slender^2^. The metatarsus is arctometatarsalian, with the metatarsal III pinched between metatarsals II and IV proximally, as in all tyrannosaurids^2,19^, and some non-tyrannosaurids such as *Appalachiosaurus*^37^, *Bistahieversor*, *Dryptosaurus*^46^, and *Raptorex*^38^. In contrast, a more normal theropod metatarsus, in which metatarsal III is large and un-pinched, is seen in the early branching tyrannosauroids, *Kileskus*^7^, *Dilong*^21^, *Eotyrannus*^47^, *Guanlong*^20^, and *Yutyrannus*^22^. The pinched proximal shaft of the metatarsal III is visible in anterior view. The whole preserved portion of metatarsal III is unobservable in posterior view, with metatarsal III and IV almost contacting each other. This condition where metatarsal III is proximally constricted is observed in tyrannosaurids and some non-tyrannosaurid tyrannosauroids. However, the proximal end of metatarsal III does not reach the proximal articulate surface with the tarsus, most likely due to poor preservation. The right metatarsal III is exposed in medial view, though its proximal portion missing. The preserved proximal portion is narrow in medial view, and it flares smoothly distally where it reaches its great width around the midpoint of the preserved shaft. The left surface shaft is inflated. The shaft has a constricted neck just proximal to the condyle. The medial ligament pit is large, deep, circular, and well-developed on the medial side of the condylar region.

Some nondescript pedal phalanges are preserved disarticulated and located beside the right metatarsus, with one exposed in dorsal view and two exposed in lateral view. They are considered to be from the right pes since they are non-ungual phalanges. In the dorsal exposed phalanx, it is constricted in the middle, with expanded proximal and distal articular regions.

## Discussion

**Figure 8:**
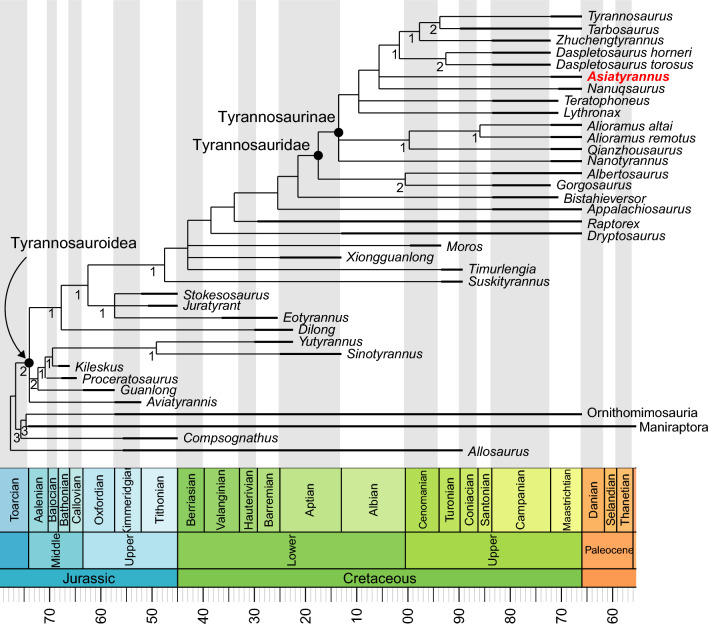
The strict consensus tree produced by phylogenetic analysis. Values beneath nodes indicate Bremer support.

The phylogenetic analysis resulted in the recovery of 8 MPTs, (tree length = 861; consistency index = 0.521; retention index = 0.793), the strict consensus of which places *Asiatyrannus* in the most derived tyrannosauroid clade–Tyrannosaurinae (Fig. 8).

*Asiatyrannus* is a tyrannosaurid theropod based on its possessing the following features: four incisiform premaxillary teeth, fused nasals, infratemporal fenestra constricted by the squamosal-quadratojugal flange, and the arctometatarsalian foot^2^. Both *Asiatyrannus* and *Qianzhousaurus* are discovered in the Nanxiong Formation of Nankang District, Ganzhou City^10^. *Asiatyrannus* differs from *Qianzhousaurus* with a proportionally deeper snout, longer premaxilla, deeper maxilla, and deeper dentary, and the cornual process of the lacrimal is inflated without a discrete horn^10^. Generally, the skulls of tyrannosauroids become deeper as their size increases, however, the *Asiatyrannus* skull (47.5 cm) is only around half in length as *Qianzhousaurus* (90 cm) but is proportionally deeper. *Asiatyrannus* is the first definitive deep-snout tyrannosaurid dinosaur from southern China and is the southernmost occurrence of tyrannosaurids in Asia.

The phylogenetic analysis places *Asiatyrannus xui* as a derived tyrannosaurid deeply nested in Tyrannosaurinae, positioned apart from the long-snouted alioramins and other early branching tyrannosaurines, and closer to the largest deep-snouted *Tarbosaurus*, *Tyrannosaurus*, and *Zhuchengtyrannus* (Fig. 8). In the latest Cretaceous, tyrannosaurids became restricted to Asia and North America, and they developed into large-bodied, bone-crunching apex predators—as exemplified by *Tarbosaurus*, *Tyrannosaurus*, and *Zhuchengtyrannus*^4,33,36^. Among tyrannosaurids, ‘*Nanotyrannus*’ (skull length 57.2 cm) and *Nanuqsaurus* (estimated skull length 60–70 cm in mature individuals), both larger than *Asiatyrannus*, might also represent the small to medium-bodied tyrannosaurids^48,49^. However, the taxonomic status of ‘*Nanotyrannus*’ is highly controversial, with the interpretation of ‘*Nanotyrannus*’ as a juvenile *Tyrannosaurus* being widely accepted^1,30,48,50^. Thus *Nanuqsaurus* might be the small-bodied tyrannosaurid that inhabited in high-latitude of North America^49^. In seven of our eight MPTs, *Asiatyrannus* and *Nanuqsaurus* were recovered as sister taxa, however, only one character of the small body size (char 1) grouped them together. New material from the Prince Creek Formation suggests *Nanuqsaurus* has an adult body size more closely comparable to other North American tyrannosaurid taxa, such as *Albertosaurus*^51^. Thus, *Asiatyrannus* currently represents the only definitive small to medium-bodied tyrannosaurine.

More than one tyrannosaurids were discovered in several well-sampled assemblages, such as the Dinosaur Park Formation, the Judith River Formation, and the Nemegt Formation^1^. *Asiatyrannus* is part of the diversification of deep-snouted tyrannosaurids and emphasizes the higher species richness of theropods, especially tyrannosaurids, in southeastern China. The discovery reveals the coexistence of the long-snouted and deep-snouted tyrannosaurid in southeastern China as in the Late Cretaceous of central Asia^27^. However, the body size of the deep-snouted and long-snouted tyrannosaurids is the opposite in southeastern Asia compared to central Asia. *Asiatyrannus* is a small to medium-sized tyrannosaurine, with an estimated total body length of approximately 3.5–4 m. The histological study demonstrated that the individual is not a fully growth adult, however, *Asiatyrannus* is less than half the size of the other large-bodied tyrannosaurines in similar growth stages. Also, *Asiatyrannus* is only half the size of the coeval long-snouted *Qianzhousaurus*^10^. *Asiatyrannus* and *Qianzhousaurus* have different skull proportions and body sizes, suggesting they may occupy different ecological niches. In the Campano-Maastrichtian of eastern/central Asia and Laramidia, the large carnivore guilds are monopolized by tyrannosaurids, with adult medium-sized predators rare or absent^52^. Holtz^52^ interpreted the “missing middle-sized” niches in the theropod guilds of Late Cretaceous Laramidia and Asia may have been assimilated by juvenile and subadults of tyrannosaurid species. In southeastern China, the *Qianzhousaurus* undoubtedly occupied the apex predator^10^, but *Asiatyrannus* might represent the small to medium-sized theropod niche between the large-bodied *Qianzhousaurus* and the diversified small-bodied oviraptorosaurs.

## Methods

### Histological sectioning

To assess the terminal age, growth rate, and ontogenetic stage of the holotype of *Asiatyrannus xui* (ZMNH M30360), we processed a histological thin section from the mid-diaphyseal of the right fibula. We prepared the histological thin section in the Key Laboratory of Vertebrate Evolution and Human Origin of Chinese Academy of Sciences, Institute of Vertebrate Paleontology and Paleoanthropology, Chinese Academy of Sciences (IVPP). The section methodology followed the procedure described by Zhao et al.^53^. The samples were impregnated with resin and placed in a vacuum chamber to reduce the formation of air bubbles for approximately 12 h and were then embedded in Technovit 7200 VLC light curing 1-component-resin, and cut transversely at the mid-diaphysis of the limb bones. Sectioned surfaces were smoothed with grinding powder and afterward glued onto a glass slide with Araldite and ground to a thickness of 60 to 100 μm. Finally, a glass cover slip for sample protection. The sections were examined in normal light and cross-polarized light and imaged using the Zeiss Axio Imager 2 MAT Upright Microscope with Zen pro 3.7 software in IVPP.

### Phylogenetic analysis

To determine the systematic position of *Asiatyrannus xui*, we used the dataset that focused on the tyrannosauroid evolution^54^, which is based on the dataset of Brusatte and Carr^3^. We added *Moros*^25^, *Suskityrannus*^33^, and *Asiatyrannus* to the dataset for analysis. Although the status of ‘*Nanotyrannus*’ currently remains unresolved, we added it to our analysis. and we coded ‘*Nanotyrannus*’ following Longrich and Saitta^1^. The revised dataset includes 37 taxa and 386 characters. We assembled the dataset in Mesquite V3.81^55^, and analyzed the dataset in TNT version 1.6 under maximum parsimony^56^, with 49 characters (1, 6, 7, 13, 14, 15, 18, 24, 34, 37, 42, 50, 53, 73, 82, 83, 98, 103, 104, 110, 116, 126, 132, 133, 148, 155, 159, 160, 161, 209, 240, 241, 244, 251, 257, 259, 264, 283, 290, 305, 307, 323, 345, 353, 359, 361, 362, 366, 383) ordered following Carr et al.^54^. We first analyzed the data matrix under the ‘New Technology’ search options, using sectorial search, ratchet, tree drift, and tree fuse options with default parameters. The stored trees were then analyzed in ‘Traditional Analysis’ under the tree bisection reconnection branch (TBR) swapping algorithm.

### Nomenclatural acts

This published work and the nomenclatural acts it contains have been registered in ZooBank. The LSIDs are D0B4FE1C-F558-4A0D-B524-20191D890A9B for this publication, 0248BA77-3106-4E7F-83A4-C67EA6777A54 for the new genus *Asiatyrannus*, and BE376950-B55A-40FE-8A1D-5E5081803422 for the new species *Asiatyrannus xui*.

### Supplementary Information

Supplementary Information.

## Data Availability

Data is provided within the manuscript or supplementary information file.
