# Supplementary material for: The first deep-snouted tyrannosaur from Upper Cretaceous Ganzhou City of southeastern China
Source: Sci Rep. 2024 Jul 25;14:16276. doi: 10.1038/s41598-024-66278-5 (PMC11272791; doi:10.1038/s41598-024-66278-5)
Supplement: Supplementary file 1 — Supplementary Information. [file 41598_2024_66278_MOESM1_ESM.docx]

Supplementary Information:

**The first deep-snouted tyrannosaur from Upper Cretaceous Ganzhou City of southeastern China**

Wenjie Zheng^[[1]](#footnote-1)^, Xingsheng Jin

Zhejiang Museum of Natural History, Zhejiang 310014, P.R. China

Text S1. Data matrix in TNT format.

xread

386 37

Allosaurus 101000210000000000101000000000000100000010000110000000000000002101110000100100100010010001???0?0000000000010?0??0100?0000000?000000000000000000?11000001000000000000?0?01000000000000????000?0001000?00100?00000001000?00000000000000000000000000200000000000000000000000010000010110000002001000[0 1]0000100000000010000000000001001010?000000010001??001000?0001010001000000010200111000000000001010

Maniraptora [0 1]000000000?000000[0 1]00000000?000000100?00?000011100[0 1]0000000001000000??1??00[0 1]00000?01000?01?00??0?0000000000000?0??0010?0010000?00001000001010010[0 1]000????00000000000000?0?00??0[0 1]000??001000?0?0?0000002[0 1]1[0 1]000?1000[0 1]010000?00000000[0 1][0 1]0000000000000000001[0 1]0000000?0000000[0 1]0001000000[0 1]00[0 1]100001?111100[0 1]1000?000000000000100000000000000000?000000000000??100001100??011000000000100001000000100000000000

Ornithomimosauria [0 1]00000000001000000?00000?1?000000000?00?0000100000??00000001?00000??1??00000000?0210??00?00000?0000001000000?0???010?0000?00??0000000?01000000[0 1]000??00000?0000?00000?0??0?00?0??00001000?????0?00002000010?00000000[0 1]00??????0011000000???0001000000000000000?0??00000?10?000?00?00000000000000100[1 2]00000000000000001000000[0 1]000[0 1]010100?010010010[0 1]00??000000?0[0 1]00011001100000000000000000100100?00?00

Compsognathus 0?000100000000000000000010?000000100?00?0?000000?1?00000000??02000??1???000000??0010??00?0?????000000000000?10???010?1000??0??000??0?000?0?0??10????0000??0000?0?000?0????0???????00?????????0???????????????????????????????????0?000000000[0 1]?000000???0?????0??0000[0 1]0[0 1]0?0??????0?0?0000??00000?000?0?0??0000010000?0??00100?[0 1]000000?0[0 1]0?0?010000??0?0000?00?0010000?0?00?00????0???00?000?0????00

Proceratosaurus 000?12000001101112?01000?0?001000100?00?1?00000010???01????0?0?????????????????????????0?10000?000001010000?1??????0???0???0?0?????100000000000???0??0?????????????????????0?000??000?????0??0?0?????????????00?100??????????????0001???110?1?000000001000???0??111000101?????????????????????????????????????????????????????????????????????????????????????????????????????????????????????????

Kileskus 0???12?????110111200100??0?00?000?00?0000?0?000??000??????????????????????????????????????????????????????????????????????????????????????????????????????????????????????????????????????????????????????????????????????????????????????????0100010???????????????0??0????????????????????????????????????????????????????????????????????????????????????????????????????????????????000??????0

Sinotyrannus 0???12??????10?1?1?1110??0?0??00?????0??1?0?0?????????1??????????????????????????????????????????????????????????????????????????????????????????????????????????????????????????????????????????????????????????????????????????????????1???????????????????????????0?01???????????????????????????????????????????????????????00?100?0??1010001000??????????????????????????????????????????????

Yutyrannus 10101221?101101111?11002?0?000000100??1?1?0?000010????110??000?011111000??0000??0210???0?1000???0?011121?01?12100002?0000??1????00?20?00?0?0????????1???????11?2???10?101?????????0010000????????????????????????????????????????01010?0?11?1?01020200100???0?0?11?00000?000?00??01?0100???00100?1????1000000000000?0011?000?00000010??10010110000000??011?0?101001001?01??1???0[0 1]00010??00?00?0?00

Guanlong 000012?00000101112001000?0?00100?100?00?1000000?10?0?010000000?0012?1?00110000???010??00?100011000001010000?10???010?1100000?0000001000000000?0?00??0000000011?0000100000?0??00000?????????????02001?0?010??0000100000?000??0????0000????10?1?0000000010?????0??101010?010????????00000001000001010000?0?0000001000000100000000000010000001010000000001010000000000001?011000000000010000000?00?00

Dilong 000002000001101002?01001?0?001000000100?1?00000000??100000000001012?1?00100100??0010??000100011100001010000?10???000?1110000??000001000000000?100?????00000000?0000101000?0????????????????????02001?0?0100?0?0010?0?????????????01001??100?1?010002?00?11???0??10101000100000010000000001000000????????000000110000001???0??0100001000?0??0?0001110??10??00010??000???011?0000000000010000??01?00

Eotyrannus 0???0??????11?100101110??????????????00?0?0?1???????2?01010000?101001?001??1??0???000?00????????????????????0???????????????????????????????0011?1???0????????????????????????????001??????0?00?????????????????????????????????????000?10000?02110100??????????2?2??0?010???00?0?0???00???1?0???????????00???011011011??0000???00?11????????????11????????????????????????????????01???000?001?00

Juratyrant 0????????????????????????????????????????????????????????????????????????????????????????????????????????????????????????????????????????????????????????????????????????????????????????????????????????????????????????????????????????????????????????????????????????0????????000?00?10000100?00100?0?????????????????????????11100000101000111111??10001100100101??????110000001?????????????

Stokesosaurus 0??????????????????????????????????????????????????????????????????????????????????????????????????????????????????????????????????????????????????????????????????????????????????????????????????????????????????????????????????????????????????????????????????????????????????????????????????????????????????????????????????110?0001010001001111????????????????????????????01?????????????

Aviatyrannis 0??????????????????????????????????????????????????????????????????????????????????????????????????????????????????????????????????????????????????????????????????????????????????????????????????????????????????????????????????????????????????????????????????????????????????????????????????????????????????????????????????100?0000010000011101?????????0???11????????????????????????????

Timurlengia 00?????????????????00?01??????10??10??1?0?001?1?0101??????????????????????????????????????????????????????????????????????????????????????????0???????010?21001??1?????????????????????????????02?0201?01001000??0????01000??0111??????????1??11??01????????????2?2?0??1?0????????????????00011?????????????????????????????????00???????????????????????????????????????????????????????????????1

Xiongguanlong 0001000000?1102001????0??0???1?0??00?01?0?001?0?01??1?0001010??001001?001?0100???000??0??0??????0???0111000??0??0?00?0110000???000?21?111110100?010?1??????100?10?1111?0??10?0?100????????????001002?00110??000?10?1????????????????????????????????????????????2?2?01?0?0??00001001000110000010??????????????????????????????????110?101????001111?0?????????????????01110012?0??????????????????

Dryptosaurus 1?????????????????????????????????????????00?????????????????????????????????????????????????????????????????????????????????????????????????????????????????????????????????????????????????????????????????????????????????????????????0?0??1212?1?1???????????????1?00???????????????????????????????????????10100?????1?????11?????????????????????1???????12?????????1012??1??011111?????1???

Appalachiosaurus 1??1?0?????????????000001001??000100?01?01001111???1200101?1?1?001101?002?01010???1?0?0??????????????????????????????????????????????????????????????????????????????????????000000010100010?00??????????????????????????????????11?110??01?1??????????0??1?????2?2?0?0????????????????????????????????0???????????????????????????????????????????????1???????12111?1111110????1111111111110110?0

Bistahieversor 1?0?0011010111200110000010?101000210101?0100111102??200101011010011001102000010?02110??1000000?00?0001210001021100010001000001??10?311111110??1??10211010021001221111110101??00000?010001110??????1???11?111111[0 1]101?1110110011?1?1111100?011001212101100112?1?0?2?2?0100?????111???????????????????????0111111????????????????????????????????????????????????????????111?1????101110111111?110001

Gorgosaurus 10110011010111200110000010011[0 1]1002001[0 1]100100111102112001010111100111011021[0 1]1111002110001000001010101012101010200011100?100010??110?310111110111111121101012100112111111010101001000111[0 1][0 1]1110?00110121111011111101111111011001????11111001011101212111100?12?1?0?21210?0101??????1???11????????1?????????01111110212101111111?0101121011111010111010000?1211111012111?021???1???1111111111111112001

Albertosaurus 101100110101112001?0000010010[0 1]1002001010010011110211200101011120011101102001110?02110001000[0 1]010101010021010102000111000100010?011003101111101?1?111?1101002100111111111010100001000111111110?00110121111011111101111111011001111111111001011101212111100112?100?2121010101??????1?0111????11101?????1?0??11111102121010111111010112101111101001101000011211111012111?0111?11?2??111111111111112001

Lythronax 1????0???1?????????00000?0010?00??10?01101101?1102112?011001?1?0?????????????????????1???001010?02010121000?0??????????????????????????1?11?11?1?1011?0100211?1?11?111????????????????????????????????????????????????????0?1??????1?????01?0?1222?211??????1???????1?0?????????????????????????????????1???????????????????????????????????0??????????121111?11?????????????????11?111?1??????0??

'Alioramus_remotus' 000?0????1????????????0??????????1?0??1?0?001????2??2?0111?1???0?????????????????????????????????????????????10000?1001?0??11??1?1?3??10?1?01???1?????01???1001?21111110101????????????????????1101211?1011?11111011?1?1???0?????01??????0??1?121???????????????????0?1?????????????????????????????????????????????????????????????????????????????????????????????????????????????????1?????????

'Alioramus_altai' 00010011?11????????00000011111100101111101001111021121011101012011110000210112101111010111000110010101220001010000010011001112011113201111101111110111010021001121111110101??101110011011101000110121111011111111011011111001111101111001011101212111110??100000????0?10000010001000111110?11011?21011111??????????????????????????10?111101?0110100????????????21?111?111111211???00?11111?11??01

Qianzhousaurus 000?00?10111122001100002?1?1111011011111010011110211210111010120111100102100111?1111???1?1000?1002?10122000?020000010001001112011113201111101?1?110?1101002100?1211111101010????????1????????00110???11101??11111?110??11?0?1????011100010111?121?11??1011100?00????0?11?01011??100?111?102?1011??????111111?110???????????????????0???111?100?10??000?????????????????1??1012?1111?0?11111????0??

Teratophoneus 1?????1??1??????????000010??1??????0?11?01001??102?1??????????2?011100102?01010??201010?0???????0???0121?1???201000200?1011???0?1013112111101011?1011101102111110111111?1?1????????????????????1?0121111?11110102011?11?1?0011?1?1??0100?011?01222?1???111??1???????11000?????????11?11?102???????????111????1?0211111011??????????10??1?10101?1??0000?1211111112?11101112111211?11?111???????????

Nanuqsaurus 0????????????????????????????????????????2??????????????????????????????????????????????????????????????????????????????????????????????????????????1?011??1111?21011100??1?????????????????????????????????????????????????????????110?10?10?????????????????????????????????????????????????????????????????????????????????????????????????????????????????????????????????????????????????????

'Daspletosaurus_torosus' 1111001101011220011100020001111012002111022011110211210102111120111100112110121112111111111[0 1]111012010121000102111001100101[0 1]11211111321211110111111011?0101211112211111101010110111001100111110011?1211110111111[0 1]1011111111111????11101111011011222111101112?110021210101010001111011111110211110??????1?1111111?212111?11111?111112101111101011101?0001121111111211111?12210?2?1111?1111111?11?1??

'Daspletosaurus_horneri' 111?00?10101?2?00??10002000111[0 1]0120021110220111102112101021111?01111101120100211121111111111111112??0121000?0211100110010111121111132121111110111101110?0121101221111110101011011100110011111001101211110111101?101??1?111111????1?1011110110112221211?11121110?2?21010101111?????01?11?10211110???????11???????11?1?1?????????????10??11????1?1???0?????????111????1?2122111211111111111111111111

Raptorex 0?110?1?00?111200?00000100011110000010100100111102?12?010101002001001?002?010111011010000000111000000111000000???000?0?1000???00111??011?1?0??1??1??1001??11001?21?1111????????????0?????????0??????????????????100?0???????0????010000010110?121211??0011??0???212101000000?000100100??100110??1?????0??0110000212101111?11???11110?111110000010??0?01011???1112101?1?1??1?12?10??1??111?1???????

Zhuchengtyrannus 1??????????????????1010??0?????1??00??110?111?1?0211????????????????????????????????????????????????????????????????????????????????????????????????????????????????????????????????????????????????????????????????????????????????1111?01101??????????????????????11010?????????????????????????????????????????????????????????????????????????????????????????????????????????????????????????

Tarbosaurus 111100211111222001110112001110111211301102111111021121010211012010??1?10201002111221111101101110121101211010021100021001111112111013212111101?111101101100312123201111111111?111111011011111111111121111011111002011111111011111?1111111201101122212?10111211111212111020111111?1111111?102111111??1??1?11111110212111011111?111112101111101011101000011211111112111112122111211111111111111112111

Tyrannosaurus 1111002111112220011101121011101112[0 1]0[1 3]01102111111021121010211112010??1?102010021102211111011[0 1]1110121101211011021100021001111112111013[1 2]1211111111111011111003121232011111111110111111011011111111111121111011111002011111111011111111111112011011222121101112111112121110201111110111111111021111012111111111111102121110111111111112101111101011101000011211111112111112122111211111111111111112111

Asiatyrannus 0?1?00???1?1122001?10002?0?1110?11??2?1?0?0?1??102??210111?????0?000?010??1???????????1????????????10?2110??02??001??0?????11???1??3?1?1?1??????????1?11????11?1?011111???1?????????????????????1??????10???11?????????????????????????????????222?????0????????????????????????????????????????????????????????????????????????????????????????????????????????????????00?0????????????1?????????

Moros ??????????????????????????????????????????????????????????????????????????????????????????????????????????????????????????????????????????????????????????????????????????????????????????????????????????????????????????????????????????????????????????????????????????????????????????????????????????????????????????????????????????????????????????????????????1??100?[1 2]??0?????1?1??1?01?0?

Suskityrannus ????00??????102001???001?0?0??00??0???0?0?0?11?0?0????????????????????????????????????????????????????????????????????????????????????????????????????00?0?10010???????????????????0?????????????????????????????????????????????????????0??1?021??1????11???????1?100?0?0??????????1?0111??0010??????0????????????????????????????????????????????????1??????????????00110?11?0?1101?111110?1????

Nanotyrannus 00110?110111112001-00000?0-1100??111?01?010?11?1011??0010101112001?0?1??2?01111?1??-0?00-0000?0001010?2?0-0?1100?00100?1?-1101011??310111?1?1?-111-?1101??21001??1?1111?1?10?101110-1110111--0-1101???11-1??111110110-?11-??1????11111101011??121211-110111--???21210?00??????????????????????????????????????????????????????????2101?111?1?011010000?121??11??21111??1??1?????1???????111???????

;

ccode + 0 5 6 12.14 17 23 33 36 41 49 52 72 81 82 97 102 103 109 115 125 131 132 147 154 158.160 208 239 240 243 250 256 258 263 282 289 304 306 322 344 352 358 360 361 365 382 *;

proc /;

comments 2

{36 244 ;

{36 368 ;

;

1. Corresponding author. Email: zhengwj@zmnh.com [↑](#footnote-ref-1)
